# Potential Value of Impaired Cognition in Stroke Prediction: A U.K. Population‐Based Study

**DOI:** 10.1111/jgs.14878

**Published:** 2017-04-03

**Authors:** Blossom C. M. Stephan, Kathryn Richardson, George M. Savva, Fiona E. Matthews, Carol Brayne, Vladimir Hachinski

**Affiliations:** ^1^ Newcastle University Institute of Ageing Newcastle University Newcastle upon Tyne UK; ^2^ Institute of Health and Society Newcastle University Newcastle upon Tyne UK; ^3^ School of Health Sciences University of East Anglia Norwich UK; ^4^ Department of Public Health and Primary Care Cambridge University Cambridge UK; ^5^ University of Western Ontario London Canada

**Keywords:** mild cognitive impairment (MCI), stroke, cognitive aging, cohort studies, risk factors in epidemiology

## Abstract

**Objectives:**

To determine whether the association between impaired cognition and greater risk of incident stroke is also observed when cognitive impairment is defined using different criteria for mild cognitive impairment (MCI).

**Design:**

Prospective cohort study with 10 years of follow‐up.

**Setting:**

Large multicentre study in the United Kingdom.

**Participants:**

Individuals (aged 64–105) from the Medical Research Council Cognitive Function and Ageing Study (N = 13,004). From this, a subsample of 2,640 individuals was selected based on age, center, and cognitive ability to undergo a detailed cognitive assessment.

**Measurements:**

Information on sociodemographic characteristics, health, cognition, and functional ability was collected in an interview. The Geriatric Mental State Automated Geriatric Examination for Computer Assisted Taxonomy and the Cambridge Cognitive Examination were used to determine cognitive status. Stroke incidence was derived from self‐report, informant report, and death certificates. Participants were divided into no, mild, moderate, and severe cognitive impairment according to their baseline Mini‐Mental State Examination (MMSE) score. MCI criteria were used to classify persons into four groups: no cognitive impairment, MCI, severe impairment (i.e. other cognitive impairment no dementia: OCIND) and dementia.

**Results:**

Over 10 years, 703 incident strokes occurred. Lower MMSE score at baseline was associated with greater risk of incident stroke. When cognitive status was determined according to MCI criteria, those with severe impairment (odds ratio (OR) = 1.5, 95% confidence interval (CI) = 1.0–2.2) and dementia (OR = 2.6, 95% CI = 1.6–3.4) had a significantly greater risk of stroke than those with no cognitive impairment.

**Conclusion:**

Criteria for MCI, defined using MMSE scores or clinical criteria, can capture individuals at greater stroke risk. The results highlight the need to focus on stroke risk in individuals even with MCI.

Stroke has been associated with greater risk of cognitive decline and dementia,^1^, ^2^ and impaired cognitive function has been associated with greater risk of incident stroke. Older adults (aged ≥65) with cognitive impairment or dementia have been found to have a risk of developing stroke that is two to three times as high as that of those without cognitive impairment.^3^, ^4^, ^5^, ^6^, ^7^, ^8^, ^9^, ^10^, ^11^, ^12^, ^13^, ^14^, ^15^ The association is found to be independent of stroke risk factors such as hypertension, smoking, and age. In contrast, no association has been found in younger adults (aged 48–67).^16^ Identification of individuals with cognitive impairment and at high risk of stroke would be advantageous for stroke prevention and could have important implications in terms of the management of vascular and cognitive disease.

Subjective memory complaints,^17^ in addition to impairments in global cognitive function,^3^, ^4^, ^8^, ^9^, ^10^, ^12^, ^18^, ^19^ memory,^12^, ^19^ executive performance,^5^, ^11^, ^19^ and sensorimotor skills,^20^ have all been associated with greater risk of stroke, in some but not all studies.^17^ There is also some evidence of racial differences, with higher risks for blacks than whites, especially for episodic memory, which could be due to a higher burden of vascular risk factors, higher prevalence of undiagnosed vascular cognitive impairment, lower reserve linked to less education, or genetic differences between blacks and whites, although these require further investigation.^19^ Most studies have assessed risk associated with performance on a single neuropsychological test (e.g., Mini Mental State Examination: MMSE, Trail‐Making Test, verbal fluency tasks; see^14^ for a summary of the tests used), which may be too restrictive. Further, most neuropsychological tests that have been used to explore associations, apart from the MMSE, are not typically used in clinical settings, raising questions of applicability for identifying individuals at high risk of stroke for primary or secondary prevention.^14^

Clinical criteria for mild cognitive impairment (MCI) identify individuals at risk of dementia. MCI defined using criteria for cognitive impairment no dementia has been associated with risk of stroke,^6^, ^21^, ^22^ but there are numerous definitions for MCI^23^ that vary in their inclusion criteria, and whether the association is consistent depending on how MCI is defined is not known. No study has tested whether MCI defined using the stricter, more widely applied Mayo Clinic criteria^24^ is also associated with stroke risk.

The aim of this study was to examine whether cognitive status predicts incident stroke using a population‐based framework including the link between incident stroke and cognition measured using the MMSE^25^ and the association between incident stroke and Mayo Clinic criteria for MCI.^24^

## Methods

### Participants

Data were from the Medical Research Council Cognitive Function and Ageing Study (CFAS). A description of the study has been published.^26^ In brief, random samples of individuals aged 65 and older were selected from the Family Health Service Authority lists in five areas of England and Wales: Cambridgeshire, Gwynedd, Newcastle, Nottingham, and Oxford. Equal numbers were randomly selected from each age group (65–74, ≥75) to produce an overall sample size of approximately 2,500 people in each area. Recruitment took place between 1991 and 1994. In total, 13,004 participants (aged 64–105; mean education 10.0 ± 2.3 years, range 0–34 years) completed a standardized screening interview at their place of residence. At baseline screening, information was collected on sociodemographic status, health (including self‐report of chronic conditions), and cognitive performance (measured using the MMSE and items about organic‐type mental symptoms from the Geriatric Mental State Automated Geriatric Examination for Computer Assisted Taxonomy (AGECAT)).^27^

Of the 13,004 individuals that completed the screening interview, 2,640 (20.3%) were selected based on age, center, and cognitive ability (weighted toward older individuals and more cognitively frail individuals, including those with an AGECAT rating case level of ≥3, plus all those who had incomplete MMSE scores or MMSE scores of ≤21) to complete a more‐detailed diagnostic assessment interview that included the organicity sections of the AGECAT^28^ and the Cambridge Cognitive Examination (CAMCOG).^29^ The selection strategy therefore targeted all potential dementia cases in addition to a random sample of those without dementia.

### Follow‐Up

Individuals who underwent further assessment have been re‐interviewed approximately every 2 years. Data from baseline and 2, 6, and 10‐year follow‐ups were used in this analysis (CFAS Data Version 9.0).

### Ethics

CFAS has local and multicenter ethics committee approval. All participants, or their informant, gave fully informed consent before the interview.

### Neuropsychological Evaluation

Global cognition was assessed using the MMSE. Based on MMSE scores, the following groups were defined: no (27–30), mild (24–26), moderate (19–23), and severe (0–18) impairment. These cut‐offs were derived from previous research in CFAS that found that MMSE scores ranging from 24 to 26 had high predictive accuracy for 2‐year incident dementia.^25^, ^30^

Domain‐specific cognitive function was assessed using the CAMCOG with three items excluded: items on tactile recognition of coins (10 pence, 5 pence) and calculation of their sum (omitted because UK coins had recently changed) and recognition of two people in the room (omitted because this item was originally intended for use in a hospital and is not relevant to a home setting). A total score assessing overall ability was calculated (range 0–103). Subscale scores were derived for memory (learning, recent, remote) and nonmemory (language comprehension and expression, orientation, perception, praxis, abstraction and attention and calculation) domains. Summing scores only from the memory or nonmemory subscales created composite scores. Because the CAMCOG scores were not normally distributed, impairment was defined using percentiles (the 16th centile to estimate a cut‐off score approximately one standard deviation below the mean) adjusted for age.

### Dementia

Dementia was defined as an AGECAT organicity rating case level of 3 or greater, which has been found to be similar to dementia diagnosed using the *Diagnostic and Statistical Manual of Mental Disorders, Third Edition, Revised*.^31^

### Physical Function

A modified version of the Townsend Disability Scale was used to assess activities of daily living (ADL) and instrumental activities of daily living (IADL) performance.^32^ Individuals were classified as not impaired if no help was needed with washing, hot meals, shoes and socks, heavy housework, or shopping and carrying heavy bags, and the individual could get around outside; mildly impaired if the person required regular help with heavy housework or shopping and carrying heavy bags; or severely impaired if the person was housebound or required help at least several times per week with washing, cooking, and dressing.

### Diagnosing MCI

Individuals were classified as having MCI using the following criteria^24^: no dementia; subjective or informant complaint of memory loss (defined as a positive response to one or more of the following: Have you had any difficulty with your memory? Have you tended to forget things recently? or Has he or she had any difficulty with his or her memory?); essentially preserved general cognitive function (MMSE score ≥22); no or only mild functional impairment; and, objective memory or nonmemory impairment (from the CAMCOG memory and nonmemory scores, outlined above). Individuals without dementia with normal general cognitive function (MMSE score ≥22), no or mild functional impairment, and preserved memory and nonmemory test performance were classified as having no cognitive impairment (NCI). All nonnormal individuals who failed to fulfill criteria for MCI (e.g., MMSE score <22, severe functional impairment and no dementia) were classified as having other cognitive impairment no dementia (OCIND). A detailed description of the OCIND group has been published previously.^33^

### Stroke Assessment

Stroke incidence was determined from three sources: self‐report and informant report at each interview and death certificates. Participants were asked whether they had ever had a stroke that required medical attention. A stroke was reported from the death certificate if an *International Classification of Diseases, Ninth Revision*, code of 430–438 was recorded. Based on the follow‐up interviews, three waves were defined to map stroke incidence over time: Wave 1 (baseline to 2‐year follow‐up), Wave 2 (2‐ to 6‐year follow‐up), and Wave 3 (6‐ to 10‐year follow‐up). This ensured that there were sufficient numbers of respondents at the end of the wave with incident stroke. To ascertain incident stroke, at the start of each wave, those reporting a history of stroke were excluded. Individuals with incomplete stroke history were also excluded. A full description of the CFAS interview schedule and the definition of each wave are shown in Figure S1 and Table S1.

### Covariates

Information on sex, age, education (<10 vs ≥10 years), and vascular risk factors (self‐reported history of heart attack, diabetes mellitus, hypertension, angina pectoris, and smoking status (never, past, current)) was collected at each interview wave.

### Statistical Analysis

All analyses were completed using Stata version 14 (Stata Corp., College Station, TX). Participants reporting a history of stroke at baseline or with missing cognition measures were excluded. Logistic regression was used to calculate odds ratios (ORs) and 95% confidence intervals (CIs) for the association between cognition and incident stroke over the three waves using two models: Model 1 (unadjusted for covariates) and Model 2 (adjusted for age, sex, education, wave, heart attack, diabetes mellitus, hypertension, angina pectoris, smoking status). A test for trend in ORs was also conducted. Interactions were tested between sex and the association with cognition and incident stroke.

Because of the sampling strategy and attrition during the study (excluding death), participants in each wave were back‐weighted to the population using inverse probability weighting. The weights were defined as the inverse probabilities of being included in each wave according to age, sex, education, sampling strategy, and cognitive status.

## Results

### MMSE Analysis

Detailed flow of participants included in the MMSE analysis is shown in Figure S2. Of the 11,829 participants with no history of stroke at the start of Wave 1, 2,467 (21%) were lost to follow‐up; of the 3,558 with no history of stroke at the start of Wave 2, 674 (19%) were lost to follow‐up; and of the 2,046 with no history of stroke at the start of Wave 3, 333 (16%) were lost to follow‐up. One hundred and thirty‐three (1%), 86 (2%), and 86 (4%) with incomplete stroke history and 428 (3%), 151 (4%), and 111 (5%) with incomplete MMSE scores at the start of each wave were excluded. The median time between interviews in each wave was 2.0 (interquartile range (IQR) 2.0–2.1), 3.3 (IQR 2.8–3.6) and 4.8 (IQR 4.4–5.1) years. Mortality in each wave was 11%, 20%, and 32%. Seven hundred and three incident cases of stroke were identified over the three waves (387 from interviews, 316 from death certificates). The sociodemographic characteristics and cognitive status of participants in each wave are presented in Table 1. Educational attainment was higher in groups with higher MMSE at baseline (Table S2), so it was controlled in multivariate analysis. Those who dropped out or died were older and more cognitively impaired at baseline (Table S3), and these factors are included in multivariate models.

**Table 1:** Participant Characteristics According to Wave in the Mini‐Mental State Examination (MMSE) Analysis

Characteristic	Wave 1, n = 9,078	Wave 2, n = 2,729	Wave 3, n = 1,628
	n (%)		
Start of wave			
Sex			
Male	3,781 (42)	1,079 (40)	644 (40)
Female	5,297 (58)	1,650 (60)	984 (60)
Age			
65–74	4,599 (51)	1,170 (43)	495 (30)
75–84	3,566 (39)	1,088 (40)	809 (50)
≥85	913 (10)	471 (17)	324 (20)
Education, years			
0–9	5,492 (61)	1,753 (65)	1,009 (62)
≥10	3,508 (39)	950 (35)	614 (38)
MMSE score			
27–30	5,310 (58)	1,092 (40)	807 (50)
24–26	2,260 (25)	780 (29)	404 (25)
19–23	1,164 (13)	586 (21)	303 (19)
0–18	344 (4)	271 (10)	114 (7)
Vascular risk factors^a^			
Heart attack	927 (10)	330 (12)	212 (13)
Diabetes mellitus	524 (6)	214 (8)	142 (9)
Hypertension	2,780 (31)	962 (35)	671 (41)
Angina pectoris	1,218 (14)	494 (18)	346 (21)
Smoking status^a^			
Nonsmoker	3,047 (34)	955 (35)	563 (35)
Former smoker	4,287 (48)	1,304 (48)	799 (49)
Current smoker	1,657 (18)	444 (16)	260 (16)
End of wave			
Dead	1,090 (12)	633 (23)	573 (35)
No stroke	952 (10)	541 (20)	487 (30)
Stroke	138 (2)	92 (3)	86 (5)
Alive	7,988 (88)	2,096 (77)	1,055 (65)
No stroke	7,735 (85)	2,022 (74)	995 (61)
Stroke	253 (3)	74 (3)	60 (4)

Numbers refer to those completing each wave with data on MMSE score at baseline and stroke at follow‐up.

a Percentage reported from valid responses.

For this analysis there were 246, 183, 167, and 107 incident stroke cases in the highest to lowest MMSE groups, respectively (across waves). Lower MMSE scores were associated with greater incidence of stroke (Table 2). A full multivariate model showing the effects of all covariates is shown in Table S4. The trend for all models was significant (*P* < .001). The association between MMSE score and incident stroke did not vary according to sex (OR = 0.72, 95% CI = 0.59–0.90). Participants excluded because they had missing MMSE scores at the start of each wave reported 50 incident strokes and had a significantly higher rate of stroke (OR = 2.5, 95% CI = 1.6–4.0).

**Table 2:** Association Between Mini‐Mental State Examination (MMSE) Score and Incident Stroke

MMSE Score at Start of Wave	Incident Strokes, n	Model 1		Model 2	
		OR (95% CI) *P*‐Value	Test for Trend in ORs	OR (95% CI) *P*‐Value	Test for Trend in ORs
27–30	246	1.0	*P* < .001	1.0	*P* < .001
24–26	183	1.6 (1.3–2.0) <.001		1.4 (1.1–1.8) .01	
19–23	167	2.2 (1.8–2.8) <.001		1.6 (1.2–2.1) <.001	
0–18	107	4.0 (3.0–5.2) <.001		2.2 (1.6–3.1) <.001	

Model 1: unadjusted.

Model 2: adjusted for age, sex, education, wave, heart attack, diabetes mellitus, hypertension, angina pectoris, and smoking status.

OR=odds ratio; CI=confidence interval.

### MCI Analysis

Detailed flow of participants included in the MCI analysis is shown in Figure S3. For the MCI analysis, 2,237 participants had no history of stroke at the start of Wave 1, 2,623 had no history of stroke at the start of Wave 2, and 1,483 had no history of stroke at the start of Wave 3. Twenty‐eight (2%), 84 (3%), and 68 (5%) with incomplete stroke histories and 96 (4%), 163 (6%), and 47 (3%) with missing MCI classification at the start of each wave were excluded. Of the 219 participants with MCI who died or completed Wave 1 43 had amnestic MCI, 108 had nonamnestic MCI, and 68 had mixed MCI. The percentage of participants alive but missing stroke information at the end of each wave was 27%, 25%, and 19%, leaving 1,627, 1,897, and 1,658 participants in the analysis for Waves 1, 2, and 3, respectively. The median time between interviews in each wave was 2.1 (IQR 2.0–2.2), 2.8 (IQR 2.3–3.2), and 4.8 (IQR 4.4–5.1) years. Mortality in each wave was 19%, 26%, and 37%, respectively. Three hundred and eighty‐two incident cases of stroke were identified over the three waves. The sociodemographic characteristics and cognitive status of the participants in each wave are presented in Table 3, and baseline characteristics according to MCI group are shown in Table S5.

**Table 3:** Participant Characteristics According to Wave in the Mild Cognitive Impairment (MCI) Analysis

Characteristic	Wave 1, N = 1,627	Wave 2, N = 1,897	Wave 3, N = 1,658
	n (%)		
Start of wave			
Sex			
Male	567 (35)	695 (37)	646 (39)
Female	1060 (65)	1202 (63)	1012 (61)
Age			
65–74	709 (44)	711 (37)	491 (30)
75–84	589 (36)	768 (40)	816 (49)
≥85	329 (20)	418 (22)	351 (21)
Education, years			
0–9	1,023 (67)	1,234 (66)	1,033 (63)
≥10	508 (33)	626 (34)	617 (37)
MCI			
No cognitive impairment	465 (29)	696 (37)	837 (50)
MCI	219 (13)	199 (10)	95 (6)
Other cognitive impairment no dementia	381 (23)	407 (21)	324 (20)
Activity of daily living impairment, no dementia	246 (15)	290 (15)	245 (15)
Dementia	316 (9)	305 (16)	157 (9)
Vascular risk factors^a^			
Heart attack	151 (10)	245 (13)	208 (13)
Diabetes mellitus	94 (6)	141 (7)	152 (9)
Hypertension	437 (29)	681 (36)	676 (41)
Angina pectoris	200 (13)	365 (19)	352 (21)
Smoking status^a^			
Nonsmoker	557 (37)	676 (36)	584 (35)
Former smoker	644 (43)	866 (47)	806 (49)
Current smoker	314 (21)	314 (17)	260 (16)
End of wave			
Dead	307 (19)	497 (26)	611 (37)
No stroke	266 (16)	420 (22)	515 (31)
Stroke	41 (3)	77 (4)	96 (6)
Alive	1,320 (81)	1,400 (74)	1,047 (63)
No stroke	1,263 (78)	1,349 (71)	987 (60)
Stroke	57 (4)	51 (3)	60 (4)

a Percentage reported from valid responses.

For this analysis, there were 98 incident strokes in participants with NCI, 27 in those with MCI, 79 in those with OCIND, 64 in those with ADL impairment, and 114 in those with dementia (across waves). The odds of incident stroke were greater with poorer cognitive function (*P* < .001). Participants with dementia and OCIND had significantly greater incidence of stroke than those with no cognitive impairment (Table 4; full multivariate model shown in Table S6). There was a greater incidence of stroke in the MCI group, but the result was not statistically significant. There was a potential interaction between sex and incident stroke in persons with dementia (*P* = .07). Women with dementia (OR = 3.0, 95% CI = 1.8–5.2) had a higher risk of incident stroke than men (OR = 1.2, 95% CI = 0.6–2.5). The results in men could be because of reduced statistical power to detect an effect due to the small number of men; 72% of individuals with dementia were female (Table S5).

**Table 4:** Association Between Mild Cognitive Impairment (MCI) Classifications and Incident Stroke

Classification	Incident Strokes, n	Model 1		Model 2	
		OR (95% CI) *P*‐Value	Test for Trend in ORs	OR (95% CI) *P*‐Value	Test for Trend in ORs
No cognitive impairment	98	1.0	*P* < .001	1.0	*P* < .001
MCI	27	1.2 (0.7–2.2) .46		1.6 (0.9–2.8) .15	
Other cognitive impairment no dementia	79	1.5 (1.1–2.1) .02		1.5 (1.0–2.2) .03	
Dementia	114	3.8 (2.7–5.2) <.001		2.3 (1.6–3.4) <.001	

Model 1: unadjusted.

Model 2: adjusted for age, sex, education, wave, heart attack, diabetes mellitus, hypertension, angina pectoris, and smoking status.

OR=odds ratio; CI=confidence interval.

Participants excluded because of missing MCI classification at the start of each wave reported 21 strokes. There was no evidence of an association between missing MCI classification and stroke incidence (OR = 1.4, 95% CI = 0.8–2.6).

## Discussion

This study explored the association between incident stroke and cognition stratified according to MMSE score or defined using criteria for MCI. Consistent with other studies, a significantly greater risk of stroke was found with poorer cognitive function. The results highlight the importance of identification of cognitive decline not only within the context of neurodegenerative disease (e.g., Alzheimer's disease), but also as a risk factor for cerebrovascular events such as stroke.

A greater risk of stroke was found even in the group with MMSE scores of 24–26 that persisted when adjusting for confounding factors. These results are consistent with previous findings linking impaired global cognitive function with major cerebrovascular events such as stroke and suggest that poor MMSE performance is a marker for covert cerebrovascular disease.^9^, ^11^ These findings highlight the potential of even subtle, preclinical cognitive deficits as a possible risk factor for stroke.

In contrast, when cognitive impairment was defined using MCI criteria, significant effects were found not in participants with MCI but in those that these classifications excluded (e.g., OCIND and dementia). There was also greater risk of stroke in the MCI group, albeit not statistically significant, that suggests that the size of the association was not large and that the statistical power of the study was too limited to detect small effects. Additional studies in larger cohorts of individuals with MCI with more stroke cases are needed to replicate and extend these findings.

The mechanisms underlying the link between cognitive impairment and risk of stroke are poorly understood. It has been hypothesized that cognitive decline may represent early manifestation of vascular disease (or subclinical cerebrovascular injury)^11^, ^12^ or poor vascular control (e.g., medication use) and risk factor reduction (e.g., physical activity) in individuals with impaired cognition.^6^ The analyses were adjusted for demographic, lifestyle, and comorbidity variables, and although associations were attenuated, they remained significant. This suggests that the associations were independent of the underlying health status and the health risk behaviors controlled for in the current analysis.

### Strengths and Limitations

In CFAS, stroke was self‐reported, so incidence estimates may reflect bias in memory or awareness of the condition, but previous studies have found that self‐report is reasonably accurate in estimating stroke in cohort studies.^34^ As with any longitudinal study of aging, there is missing data because of death and attrition, which can lead to selection bias. All results were therefore weighted for study design and attrition. Cognition was measured using the MMSE, and although the MMSE has limitations (e.g., floor and ceiling effects), it is one of the most widely used cognitive screening tools in clinical and research practice. It has also been found to perform as well as other MCI definitions in predicting 2‐year risk of incident dementia in CFAS.^25^ Therefore, the current study investigated whether different MMSE score categories are associated with risk of stroke. In addition, the associations were tested using more clinically based criteria, including the definition for MCI. Neither pure amnestic nor nonamnestic MCI were investigated because they are rare in CFAS and therefore have limited statistical power.^35^

## Conclusion

It is possible that individuals with cognitive decline could be benefit from careful screening and management of vascular risk factors to prevent occurrence not only of dementia, but also of stroke. An opportunity for further research is the development of accurate models for stroke risk prediction in individuals with cognitive impairment so that high‐risk individuals can be targeted for stroke prevention.

## Supporting information


**Appendix S1.** Extended Description of the Study Design, Characteristics of Participants and Results.
**Figure S1.** Medical Research Council Cognitive Function and Ageing Study Design.
**Figure S2.** Participant Flow Through the Mini‐Mental State Examination (MMSE) Score Analysis.
**Figure S3.** Participant Flow Through the Mild Cognitive Impairment (MCI) Analysis.
**Table S1.** Medical Research Council Cognitive Function and Ageing Study Interviews Defining Start and End of Each Wave According to Cognitive Analysis.
**Table S2.** Educational Attainment Stratified According to Baseline Mini Mental State Examination (MMSE) Score.
**Table S3.** Demographic Characteristics and Mini‐Mental State Examination (MMSE) Scores of Participants Starting Wave 1 of the MMSE Analysis Stratified According to Status at End of Wave 1.
**Table S4.** Full Multivariate Logistic Regression Model for Incident Stroke Given Baseline Mini Mental State Examination (MMSE) Score Group and Covariates.
**Table S5.** Demographic Characteristics and Mini‐Mental State Examination (MMSE) Scores of Baseline Sample Stratified According to Mild Cognitive Impartment (MCI) Status (Wave 1).
**Table S6.** Full Multivariate Logistic Regression Model for Incident Stroke Given Baseline Mild Cognitive Impairment (MCI) Status and Covariates.
