# Supplementary material for: Potential Value of Impaired Cognition in Stroke Prediction: A U.K. Population‐Based Study
Source: J Am Geriatr Soc. 2017 Apr 3;65(8):1756–62. doi: 10.1111/jgs.14878 (PMC5574015; doi:10.1111/jgs.14878)
Supplement: Supplementary file 1 — Appendix S1. Extended Description of the Study Design, Characteristics of Participants and Results. Figure S1. Medical Research Council Cognitive Function and Ageing Study Design. Figure S2. Participant Flow Through the Mini‐Mental State Examination (MMSE) Score Analysis. Figure S3. Participant Flow Through the Mild Cognitive Impairment (MCI) Analysis. Table S1. Medical Research Council Cognitive Function and Ageing Study Interviews Defining Start and End of Each Wave According to Cognitive Analysis. Table S2. Educational Attainment Stratified According to Baseline Mini Mental State Examination (MMSE) Score. Table S3. Demographic Characteristics and Mini‐Mental State Examination (MMSE) Scores of Participants Starting Wave 1 of the MMSE Analysis Stratified According to Status at End of Wave 1. Table S4. Full Multivariate Logistic Regression Model for Incident Stroke Given Baseline Mini Mental State Examination (MMSE) Score Group and Covariates. Table S5. Demographic Characteristics and Mini‐Mental State Examination (MMSE) Scores of Baseline Sample Stratified According to Mild Cognitive Impartment (MCI) Status (Wave 1). Table S6. Full Multivariate Logistic Regression Model for Incident Stroke Given Baseline Mild Cognitive Impairment (MCI) Status and Covariates. [file JGS-65-1756-s001.docx]

Appendix 1. Extended description of the study design, characteristics of participants and results. The Appendix presents an illustration of the Medical Research Council Cognitive Function and Ageing Study interview schedule and shows how the different interviews were combined to form waves for the Mini-Mental State Examination and mild cognitive impairment analyses. Three waves were defined such that each wave consisted of interviews no more than 5 years apart to ensure sufficient numbers of respondents at the end of the wave in the low cognition groups and adequate stroke recall. Participant flow through each study is also shown.

Figure S1. Medical Research Council Cognitive Function and Ageing Study design.

**
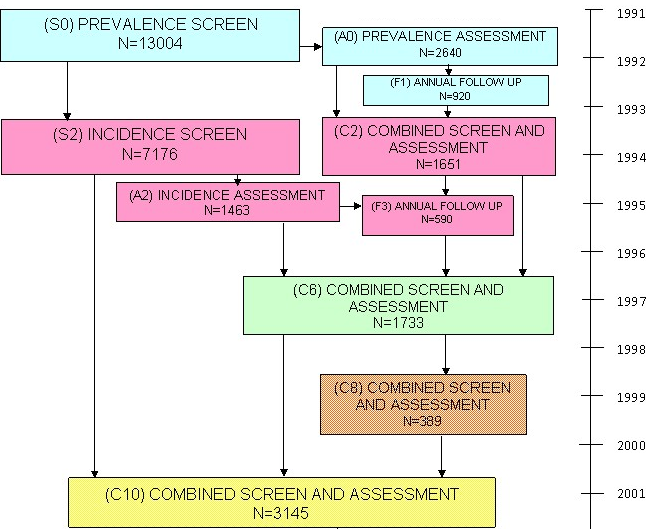
**

Figure S2 Participant flow through the Mini-Mental State Examination (MMSE) score analysis.

Figure S3 Participant flow through the mild cognitive impairment (MCI) analysis.

Table S1. Medical Research Council Cognitive Function and Ageing Study Interviews Defining Start and End of Each Wave According to Cognitive Analysis

| Wave | Mini Mental State Examination Analysis | | Mild Cognitive Impairment Analysis | |
| --- | --- | --- | --- | --- |
|  | Start | End | Start | End |
| 1 | s0 | s2 or c2 | a0 | c2 |
| 2 | s2^a^ or c2 | c6 or s6 | a2 or c2 | c6 |
| 3 | c6 or s6 | cx | c6 | cx |

^a^Including only those from s2 going to the assessment arm (a2 then c6) or those in Cambridgeshire (going on to s6)

Table S2 Educational Attainment Stratified According to Baseline Mini Mental State Examination (MMSE) Score

| Educational Level, Years | Baseline MMSE Score | | | | |
| --- | --- | --- | --- | --- | --- |
|  | 27–30 | 24–26 | 19–23 | 0–18 | Missing |
|  | n (%) | | | | |
| 0, not known | 11 (0.2) | 12 (0.4) | 24 (1.4) | 76 (14.6) | 65 (24.4) |
| <9 | 264 (4.2) | 225 (7.5) | 230 (13.1) | 73 (14.1) | 31 (11.7) |
| 9–10 | 3,152 (50.2) | 1,824 (60.6) | 1,113 (63.3) | 274 (52.8) | 113 (42.5) |
| ≥11 | 2,851 (45.4) | 948 (31.5) | 390 (22.2) | 96 (18.5) | 57 (21.4) |

Table S3. Demographic Characteristics and Mini-Mental State Examination (MMSE) Scores of Participants Starting Wave 1 of the MMSE Analysis Stratified According to Status at End of Wave 1

| Characteristic | Completed, n=8,173 | Died, n=1,189 | Dropped Out, n=2,467 |
| --- | --- | --- | --- |
| Female, % | 59 | 53 | 67 |
| Age, median (IQR) | 74 (69–79) | 80 (74–85) | 74 (69–80) |
| MMSE score, median (IQR) | 27 (25–29) | 25 (20–27) | 25 (22–28) |

IQR=interquartile range.

Table S4. Full Multivariate Logistic Regression Model for Incident Stroke Given Baseline Mini-Mental State Examination (MMSE) Score Group and Covariates

|  | Odds Ratio (95% Confidence Interval) |
| --- | --- |
| MMSE score (reference 27–30) |  |
| 24–26 | 1.42 (1.10–1.84) |
| 19–23 | 1.64 (1.25–2.15) |
| 0–18 | 2.21 (1.56–3.13) |
| Missing | 2.55 (1.59–4.07) |
| Age (per year) | 1.07 (1.05–1.08) |
| Female | 0.72 (0.59–0.90) |
| Education, years (reference ≥11) |  |
| 9–10 | 0.62 (0.24–1.60) |
| <9 | 1.01 (0.73–1.40) |
| Missing | 0.98 (0.79–1.20) |
| Smoking (reference nonsmoker) |  |
| Former smoker | 0.93 (0.74–1.16) |
| Current smoker | 1.36 (1.02–1.81) |
| Previous heart attack | 1.10 (0.80–1.50) |
| Diabetes mellitus | 1.32 (0.96–1.79) |
| High blood pressure | 1.41 (1.16–1.71) |
| Angina pectoris | 0.95 (0.73–1.25) |
| Wave (reference 1) |  |
| 2 | 1.07 (0.88–1.31) |
| 3 | 1.70 (1.37–2.10) |

Table S5. Demographic Characteristics and Mini-Mental State Examination (MMSE) Scores of Baseline Sample Stratified According to Mild Cognitive Impartment (MCI) Status (Wave 1)

| Characteristic | No Cognitive Impairment, n=465 | MCI, n=219 | Other Cognitive Impairment No Dementia, n=318 | Dementia, n=316 | Activity of Daily Living Impairment, No Dementia, n=246 | Missing, n=58 |
| --- | --- | --- | --- | --- | --- | --- |
| Age, median (IQR) | 73 (69–78) | 73 (69–78) | 73 (69–79) | 84 (79–88) | 82 (74–87) | 79 (70–85) |
| Female, % | 56 | 61 | 64 | 72 | 74 | 60 |
| <10 years education, % | 52 | 72 | 78 | 77 | 66 | 77 |
| MMSE, median (IQR) | 27 (26–29) | 25 (23–27) | 23 (20–26) | 13 (0–18) | 21 (16–24) | 0 (0–18) |

IQR=interquartile range.

Table S6. Full Multivariate Logistic Regression Model for Incident Stroke Given Baseline Mild Cognitive Impairment (MCI) Status and Covariates

| Factor | Odds Ratio (95% Confidence Interval) |
| --- | --- |
| MCI status (reference no cognitive impairment) |  |
| MCI | 1.56 (0.85–2.83) |
| Other cognitive impairment no dementia | 1.50 (1.05–2.16) |
| Dementia | 2.31 (1.55–3.43) |
| Activity of daily living impairment, no dementia | 1.03 (0.69–1.55) |
| Missing MCI | 1.43 (0.77–2.64) |
| Age (per year) | 1.07 (1.05–1.09) |
| Female | 0.86 (0.65–1.15) |
| <10 years of education | 1.03 (0.78–1.37) |
| Current smoker | 1.04 (0.83–1.29) |
| Previous heart attack | 1.35 (0.88–2.07) |
| Diabetes mellitus | 1.1 (0.74–1.63) |
| High blood pressure | 1.32 (1.03–1.70) |
| Angina pectoris | 0.99 (0.70–1.41) |
| Wave (reference 1) |  |
| 2 | 1.27 (0.87–1.85) |
| 3 | 2.18 (1.50–3.16) |
